# Atlas of glomerular disease-specific genetic effects on blood transcriptome

**DOI:** 10.64898/2026.06.22.26356281

**Published:** 2026-06-24

**Authors:** Lili Liu, Chen Wang, Oleksandr Kravets, Damian Fermin, Felix Eichinger, Francesca Zanoni, Atlas Khan, Jun Y. Zhang, Yan Ouyang, Qin Li, Patrick Hamilton, Philip A Kalra, Rajkumar Chinnadurai, Kimberly Reidy, Jeffrey Kopp, Krzysztof Mucha, Cathy Smith, Abigail Smith, Michelle Mcnulty, Sean Eddy, Viji Nair, Margaret Helmuth, Bethany Klunder, Tetyana Vasylyeva, William Smoyer, Celine Berthier, Rulan Parekh, Scott Wenderfer, Tess Martin, Ksenia Solkolva, Rachel Sealfon, Chandra Theesfeld, Afshin Parsa, Rasheed Gbadegesin, Matthew Sampson, Simone Sanna-Cherchi, Olga Troyanskaya, Dirk S. Paul, Slave Petrovski, David Goldstein, Laura Heyns Mariani, Ali Gharavi, Columbia Genomics Consortium, NEPTUNE Consortium, CureGN Consortium, Matthias Kretzler, Krzysztof Kiryluk

## Abstract

IgA nephropathy (IgAN), IgA vasculitis (IgAV), focal segmental glomerulosclerosis (FSGS), membranous nephropathy (MN), and minimal change disease (MCD) account for the majority of idiopathic glomerulo-nephropathies (GN). These disorders involve immune system dysregulation and have a complex genetic architecture. Currently, there are no adequately powered blood transcriptomic datasets coupled to genetic data from patients with GN that can delineate disease-context specific genetic effects on blood immune cell transcriptome. We performed whole genome sequencing coupled with bulk blood transcriptome sequencing on 1,822 participants from the CureGN study, a prospective cohort of participants with a kidney biopsy diagnosis of primary GN. We generated disease-context specific transcriptome-wide maps of gene expression QTL (eQTL), splicing QTL (sQTL), and double strand RNA-editing QTL (edQTL) for FSGS (N=447), IgAN (N=403), IgAV (N=123), MCD (N=408), and MN (N=441), as well as cross-disease maps for all 1,822 participants. Our QTL mapping identified 16,068 eGenes, 4,644 sGenes and 4,611 edQTLs with an FDR<0.05 in at least one GN type. Approximately 5-10% of the QTL signals were unique to a specific GN type, while ∼90% were shared between at least two conditions. Colocalization analysis demonstrated that ∼80% of shared eGenes between traits also shared the same causal variants, whereas ∼2% had distinct causal variants, suggesting context-specific regulatory effects. Cross-phenotype QTL mapping uncovered 6,466 eGenes, 2,705 sGenes and 5,321 edQTLs not previously detected in GTEx. Age, eGFR, and proteinuria-interaction QTL analyses identified hundreds of loci modified by age and disease severity. Lastly, integrative analyses with GWAS nominated new candidate genes for each of the five GN types under study. In summary, we generated comprehensive maps of GN-context-specific genetic effects on blood transcriptome, providing a powerful resource for integrative gene discovery studies of primary GN.

## Full Text Availability

The license terms selected by the author(s) for this preprint version do not permit archiving in PMC. The full text is available from the preprint server.

